# Subcutaneous injection of an immunologically tolerated protein up to 5 days before skin injuries improves wound healing

**DOI:** 10.1590/1414-431X2021e11735

**Published:** 2022-02-09

**Authors:** K. Franco-Valencia, I.B.C. Nóbrega, T. Cantaruti, A. Barra, A. Klein, G.M. Azevedo-Jr, R.A. Costa, C.R. Carvalho

**Affiliations:** 1 Departamento de Morfologia, Universidade Federal de Minas Gerais, Belo Horizonte, MG, Brasil; 2 Departamento de Farmacologia, Universidade Federal de Minas Gerais, Belo Horizonte, MG, Brasil; 3 Departamento de Ciências Naturais, Universidade Federal de São João del Rei, São João del Rei, MG, Brasil

**Keywords:** Immunological tolerance, Wound healing, Skin, Collagen remodeling, Mice

## Abstract

Oral tolerance blocks the development of specific immune responses to proteins ingested by the oral route. One of the first registries of oral tolerance showed that guinea pigs fed corn became refractory to hypersensitivity to corn proteins. Mice fed with chow containing corn are tolerant to zein, and parenteral injection of zein plus adjuvant blocks immunization to unrelated proteins injected concomitantly and reduces unspecific inflammation. Extensive and prolonged inflammatory infiltrate in the wound bed is one of the causes of pathological wound healing. Previous research shows that intraperitoneal injection of zein concomitant with skin injuries reduces the inflammatory infiltrate in the wound bed and improves wound healing. Herein, we tested if one subcutaneous injection of zein before skin injury improves wound healing. We also investigated how long the effects triggered by zein could improve skin wound healing. Mice fed zein received two excisional wounds on the interscapular skin under anesthesia. Zein plus Al(OH)_3_ was injected at the tail base at 10 min, or 3, 5, or 7 days before skin injuries. Wound healing was analyzed at days 7 and 40 after injury. Our results showed that a zein injection up to 5 days before skin injury reduced the inflammatory infiltrate, increased the number of T-cells in the wound bed, and improved the pattern of collagen deposition in the neodermis. These findings could promote the development of new strategies for the treatment and prevention of pathological healing using proteins normally found in the common diet.

## Introduction

Skin injury, whether accidental or surgical, triggers an orchestrated process of cellular and molecular interactions that generally results in wound healing (1-3). The sequential progression of cellular and molecular interactions that occurs during wound healing in healthy conditions is predictable and begins with the activation of the blood coagulation cascade (hemostasis), leukocyte recruitment (inflammatory phase), keratinocyte proliferation, angiogenesis, and fibroblast activation with extracellular matrix synthesis (proliferation phase) until the remodeling of the matrix and reduction of dermal cellularity (maturation phase) (4,5).

During the inflammatory phase, the number leukocytes in the wound bed increases. Leukocytes reach the site of the skin injury through their recruitment from the blood and the areas adjacent to the lesion. Neutrophils, present in large numbers in the blood, are among the first circulating cells to arrive, followed by monocytes, which differentiate into tissue macrophages (6). Lymphocytes are also recruited to the wound bed and their numbers peak on days 7-10 after skin injury (7,8). Mast cells are abundant in healthy skin and can be recruited to the lesion area from the dermis adjacent to the lesion and from undifferentiated precursors in the blood (9,10). At the wound bed, leukocytes can phagocyte microbes and cellular debris or produce cytokines and growth factors, contributing to the restoration of tissue integrity. It is not a simple task to determine the specific function of each type of leukocyte during repair because leukocytes can have redundant, overlapping, and changing functions over time. However, excessive or prolonged inflammation may prevent lesion closure or form pathologic scars (3,11). Under normal conditions, the inflammatory infiltrate decreases after the first week, while activated fibroblasts secrete a new extracellular matrix. The reduction in the number of leukocytes in the wound area depends on the reduction in their recruitment and their death or migration away from the wound (12,13).

Skin scarring results from substitution of dermal components by an extracellular matrix (ECM) that is organized differently from the original ECM. In uninjured, intact skin, collagen fibers have a typical basket weave arrangement and are thick and densely organized. On the other hand, scars have collagen fibers arranged parallel to the epidermis and are thinner and loosely organized. Scars visible to the naked eye can range from a soft, barely noticeable line to disfiguring hypertrophic and keloid scars (14). While excessive or prolonged inflammation is related to hypertrophic scars, reduced inflammation results in scarless healing (2,15,16). Although it is known that the inflammatory phase of wound repair significantly influences its outcome, it remains difficult to predict the type of scar that will form after healing and to promote scarless healing (3,4).

Since excessive and prolonged inflammation can compromise wound closure or result in hypertrophic scarring and keloids, developing strategies to reduce inflammation or promote rapid resolution of inflammation would be useful to promote good healing in a shorter time.

It is possible to inhibit immune responses and prevent allergic and autoimmune inflammation through an immune phenomenon triggered by protein administered by the oral route, called oral tolerance (17-19). Feeding with maize (*Zea mays*) or with ovalbumin (OVA) impairs the subsequent induction of allergic reactions to zein or OVA, respectively (20,21). Oral tolerance is the systemic unresponsiveness induced specifically by protein delivered orally: feeding with zein does not impair allergy to OVA and feeding with OVA does not impair allergy to zein. However, parenteral re-exposure to an orally tolerated protein has indirect and systemic effects that inhibit immune responses to unrelated proteins injected concomitantly (22,23). Therefore, concomitant injection of zein and OVA in mice exhibiting oral tolerance to zein prevents the immune response to zein (as predictable) and to OVA - an indirect effect of oral tolerance (24). Moreover, the indirect effects of oral tolerance induced by parenteral re-exposure to a protein previously orally administered inhibit immunization to other unrelated antigens injected 3 days later, but not to antigens injected 7 days later (25). In a similar way, simultaneous injection of OVA and myelin basic protein (MBP) prevents experimental autoimmune encephalomyelitis (EAE) in mice orally tolerant to OVA (26). Furthermore, intraperitoneal injection of OVA or zein in mice with skin injuries reduces inflammation in the wound bed and improves wound healing in mice orally tolerant to these proteins but not in non-tolerant mice (27-30).

Herein, we tested if subcutaneous injection of zein in mice with skin injuries pre-treated with zein by the oral route reduces inflammation in the wound bed and improves wound healing in mice. We also evaluated how long the systemic effects of injecting a tolerized protein lasts to reduce inflammation and improve wound healing.

## Material and Methods

### Animals

Eight-week-old male C57BL/6 mice were obtained from the Institute of Biological Sciences, Universidade Federal Minas Gerais (UFMG, Brazil) and treated according to the guidelines of the Ethics Committee of Animal Experimentation of UFMG. Water and food were offered *ad libitum*. Each group had six mice per time point.

### Oral exposure to zein

All animals were fed commercial pelleted chow (Nuvilab CR-1, Nuvital Nutrientes S/A, Brazil), traditionally used for rodent feeding. The rodent chow contained corn (*Zea mays*) and zein is one of the most abundant proteins of corn.

### Subcutaneous injection of zein at the base of the tail

In a first experiment, animals in the experimental group received a subcutaneous injection of 10 µg zein (Sigma Aldrich, USA) plus 1.6 mg Al(OH)_3_ adjuvant at the tail root, just before skin injury. One control group received saline and the other control group received 1.6 mg Al(OH)_3_ adjuvant in a final volume of 100 µL. In subsequent experiments, zein was injected 3, 5, or 7 days before skin injury and control mice received saline.

### Skin injury

Mice were anesthetized with ketamine (97 mg/kg) and xilazine (16.5 mg/kg), and their dorsal thoracic skin was shaved and cleaned with 70% ethanol just before wounding. Two circular excisional injuries were made on the interscapular skin, using a biopsy punch (6 mm diameter), one on each side of the dorsal midline. The wounds were left unsutured and without dressing. After wounding, the animals were housed individually to prevent traumatic damage to the wounds by other mice.

### Macroscopic analysis

The injured skin was photographed with an in-picture ruler for scale using a digital camera (Nikon Coolpix S3100, Japan) immediately after or 7 days after injury. The images were analyzed using Image Tool software (IMAGE TOOL 3.0 UTHSCSA, USA) and wound outlines were manually traced for calculation of wound area.

### Microscopic analysis

After surgery, the animals were kept at 37°C until anesthesia recovery. Then, they were transferred to the bioterium with the temperature at 23°C and 12 h light/dark cycle. Water and food were offered *ad libitum*. On days 7 and 40 after surgery, the animals were euthanized and the wound with surrounding skin area was collected for microscopic evaluation.

### Histological analysis

The wounds on the right side of the animal were fixed in Carson's modified Milloning's phosphate-buffered formalin for 24 h, perpendicularly sectioned in half at the center, dehydrated in ethanol, and embedded in paraffin for histological studies following standard protocols. Serial 5-µm cross-sections from the middle of the wound were stained with hematoxylin and eosin (H&E), Alcian blue-Safranin, Masson's trichrome, or Sirius red. Collagen fibers stained with Sirius red were examined under polarized light (Olympus BX43 microscope, Japan) and images were captured at 200× magnification. For morphometry, mast cells were identified after Alcian blue-Safranin staining and leukocytes and fibroblasts were identified after H&E staining by their characteristic morphology at high magnification (1000×) under a light microscope (Olympus BX40 microscope). Cells were counted by two investigators in a blinded manner using an intersection grid (Thomas Scientific, USA) placed at the ocular lens. Leukocytes, fibroblasts, and mast cells were counted in 10 fields of 10,000 µm^2^ each, within the wound healing area of one section per mouse and the results from six sections per group are reported as means±SE.

### Immunofluorescence analysis

The wound on the left side of the animal was fixed in 20% dimethyl sulfoxide (DMSO) and 80% methanol at -80°C for at least 6 days. One day before processing for inclusion in Paraplast (Sigma Aldrich, USA), skin samples were transferred to -20°C and then brought to room temperature. Five-µm cross-sections from the middle of the wound were mounted on slides and submitted to standard immunofluorescence staining protocol. The following primary antibodies were used: purified rat anti-mouse CD45 (Biolegend, USA), rat anti-Mouse CD3 (BD Pharmigen, USA), and monoclonal anti-α-smooth muscle actin antibody produced in mouse (Sigma-Aldrich, USA). After 5 rinses in PBS, sections were incubated for 1 h at room temperature in the dark with the following secondary antibodies: Alexa-Fluor¯488 goat anti-mouse IgG2a or Alexa-Fluor¯488 rabbit anti-rat IgG (H+L) (Molecular Probes, USA). Nuclei were labeled with 4'6-diamidino-2-phenylindole dihydrochloride (DAPI) (Molecular Probes). Fluorescence was viewed using a laser scanning confocal microscope (LSM 880; Carl Zeiss AG, Germany) at the Centro de Aquisição e Processamento de Imagem (CAPI) of Instituto de Ciências Biológicas, UFMG. Optimal confocal settings (aperture, gain, and laser power) were determined at the beginning of each imaging session and then held constant during the analysis of all samples. To perform quantitative analysis of fluorescence, images were captured at 12 bits and analyzed in the gray scale range of 0 to 255 with Image Tool 3.0 software (http://ddsdx.uthscsa.edu/dig/itdesc.html). Fluorescence intensity was recorded as the sum of gray values of all pixels divided by the area (in μm^2^) × 10^-3^. Background fluorescence was measured in each sample and subtracted from the values obtained for the fluorescence intensity.

### Analysis of neutrophil myeloperoxidase (MPO)

One day after skin injury, mice were euthanized with an overdose of anesthetic and the wounds with about 4-mm surrounding area were removed and snap frozen in liquid nitrogen. The tissue was processed as previously described (31) and the MPO assay was performed by measuring the change in absorbance at 450 nm using 1.6 mM 3,3′,5,5′-tetramethylbenzidine (Sigma-Aldrich) dissolved in DMSO (Merck, USA) and 0.003% H_2_O_2_ (v/v) dissolved in phosphate buffer (0.05 M Na3PO4 and 0.5% hexadecyltrimethylammonium bromide [pH 5.4]). Results are reported as the relative unit that denotes activity of MPO.

### Statistical analysis

The GraphPad Prism7 program (GraphPad Software, USA) was used. The significance of the differences between experimental and control groups was determined by one-way ANOVA followed by Bonferroni test. P≤0.05 was considered significant and results are reported as means±SE.

## Results

### Effect of zein on the number of inflammatory cells and fibroblasts at the injured site

**Figure 1:**
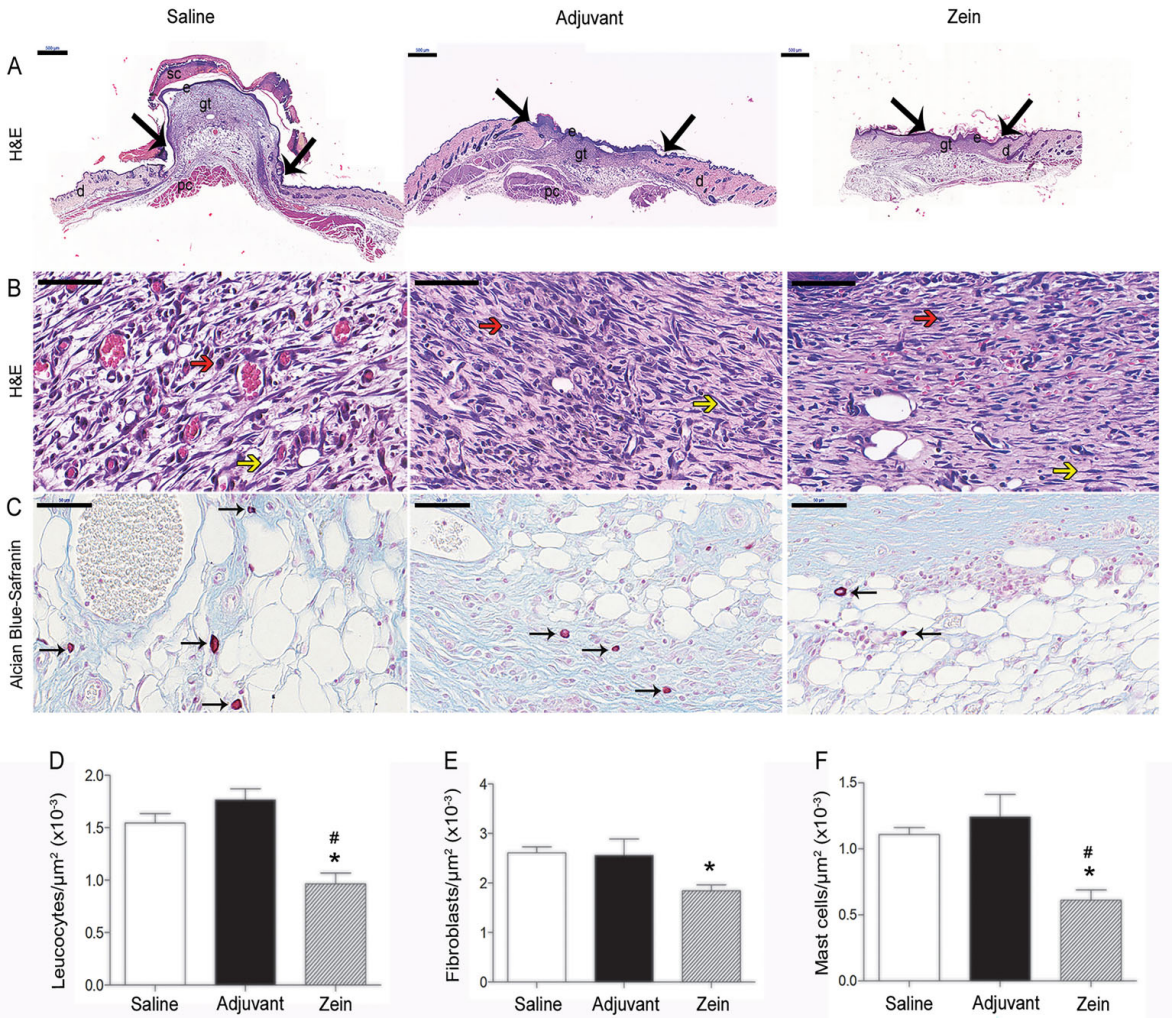
Zein reduced the inflammatory infiltrate and number of fibroblasts in the wound bed. **A**, Panoramic view of injured skin and (**B**) granulation tissue area stained with H&E or (**C**) Alcian blue-Safranin 7 days after skin injury in mice injected with either saline, adjuvant, or zein plus adjuvant minutes before injury. The arrows in **A** indicate the transition from hypertrophic epidermis to non-injured epidermis and small letters represent: e: epidermis; d: dermis; gt: granulation tissue; pc: panniculus carnosus; sc: scab. The red and yellow arrows in **B** indicate leukocytes and fibroblasts, respectively. Black arrows in **C** indicate mast cells. Scale bars: **A**, 500 µm; **B** and **C**, 50 µm. Morphometric analyses of leukocytes (**D**), fibroblasts (**E**), and mast cells (**F**). Data are reported as means±SE (n=6). *P≤0.05 compared with the saline group, ^#^P≤0.05 compared with the adjuvant group (ANOVA).

**Figure 2:**
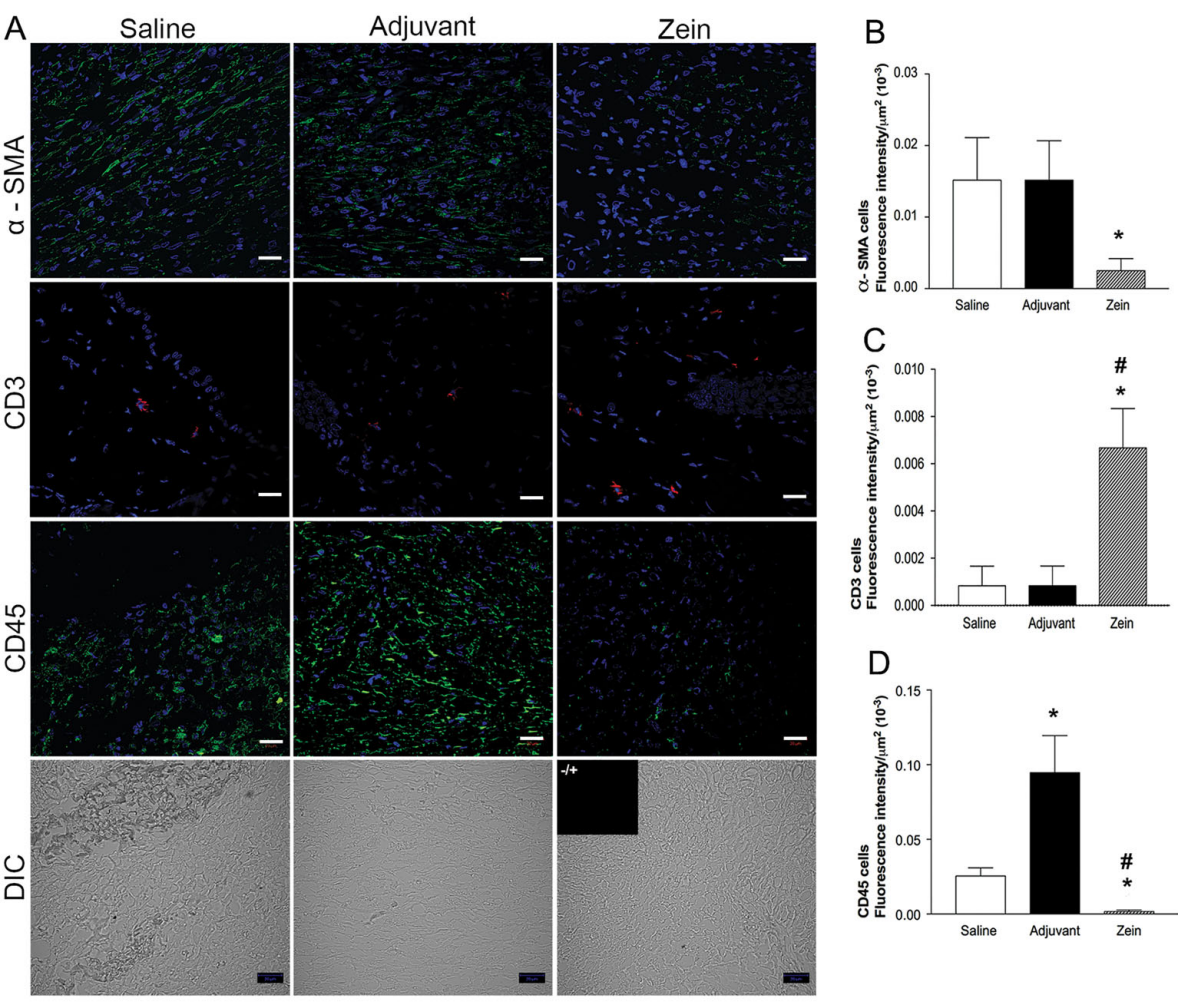
Zein increased the number of T lymphocytes in the wound bed. **A**, Representative images of skin after immunostaining with anti-α-SMA (myofibroblasts), anti-CD3 (lymphocytes), or anti-CD45 (leukocytes), and nuclear counterstaining with 4'6-diamidino-2-phenylindol (blue), 7 days after skin injury. The bottom row shows typical sections of each group using differential interference contrast (DIC) and an insert with an image of the control staining without the primary antibody. Scale bars: 20 µm. Fluorescence intensity determined in sections immunostained with anti-α-SMA (**B**), anti-CD3 (**C**), or anti-CD45 (**D**) in the saline, adjuvant, and zein groups. Data are reported as means±SE of fluorescence intensity (n=6). *P≤0.05 compared with the saline group, ^#^P≤0.05 compared with the adjuvant group (ANOVA).

Qualitative analysis of skin sections stained with H&E at day 7 after injury showed reduced inflammation in the experimental group treated with zein: there was less inflammatory infiltrate, reduced tissue edema, and less vascular congestion than in control mice (Figure 1). In mice injected with zein, the inflammatory infiltrate was more restricted to the injured site and the regenerated epithelium was less hyperplastic and more aligned with the uninjured epithelium than in control groups. Morphometric analysis of skin sections stained with H&E and Alcian blue-Safranin showed fewer leukocytes, fibroblasts, and mast cells in the wound healing area of mice injected with zein (Figure 1). Immunofluorescence analysis of skin sections incubated with anti-CD45 antibody confirmed the significant reduction in leukocyte numbers, and analysis of α-SMA expression showed less myofibroblasts in the wound bed of the zein-treated group (Figure 2). On the other hand, injection of zein increased the number of T lymphocytes in the wound bed (Figure 2).

### Effect of zein on the pattern of collagen deposition in the neodermis

**Figure 3:**
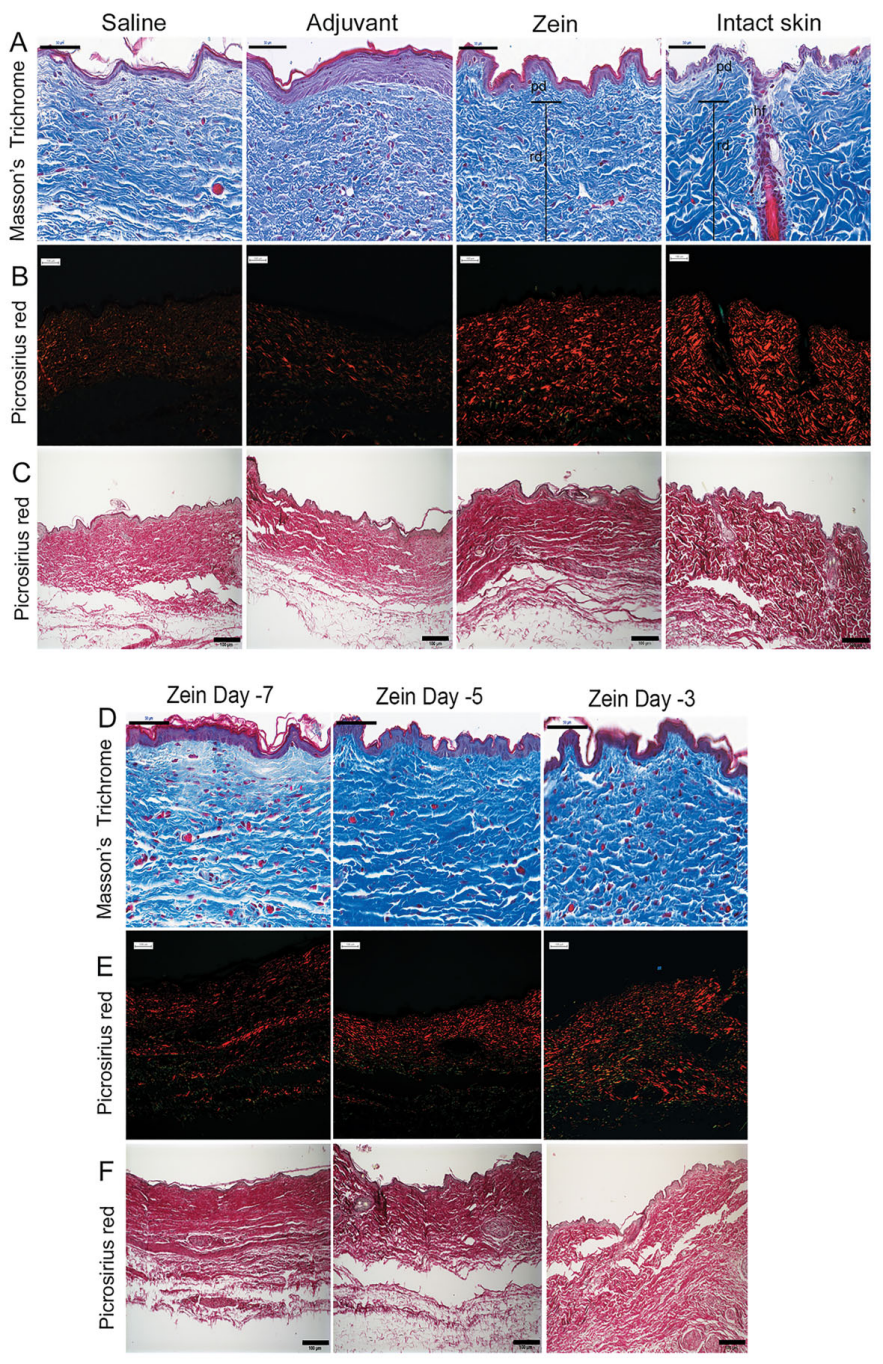
Subcutaneous injection of zein up to 5 days before skin injury improved the pattern of collagen arrangement. **A**-**C**, Representative sections of intact skin or wounds harvested 40 days after skin injury in mice injected with saline, adjuvant, or Zein plus adjuvant, minutes prior to injury. **D**-**F**, Representative sections of wounds harvested 40 days after skin injury in mice injected with Zein plus adjuvant 7, 5, or 3 days before skin injury. Skin sections stained with (**A** and **D**) Masson's trichrome, (**B** and **E**) Picrosirius red under polarization light, or (**C** and **F**) Picrosirius red without polarization light. hf: hair follicle; pd: papillary dermis; rd: reticular dermis. Scale bars: 50 µm (Masson's trichrome); 100 µm (Picrosirius red).

Figure 3 shows a representative photograph of intact, non-scarred skin stained with Masson's trichrome where one hair follicle, the papillary dermis, and the reticular dermis can be easily identified. As expected, collagen fibers in intact skin had a typical basket weave arrangement (Figure 3A) and were thick and densely organized, resulting in red-orange birefringence under polarized light microscope (Figure 3B). On the other hand, the scarred skin of the control groups had collagen fibers arranged parallel to the epidermis and with a weak birefringence, indicating a smaller thickness compared to the collagen fibers found in unscarred skin. In animals treated with zein, differently from non-treated animals, the papillary dermis was reconstituted (Figure 3A) and the pattern of collagen fibers in the neodermis was more similar to that found in intact skin, with fibers arranged in a way that resulted in red-orange birefringence under polarization light (Figure 3B).

### Effect of zein injection up to 5 days before injury on inflammation and collagen arrangement

We then determined how long the effects of zein injection lasted and improved collagen arrangement. Collagen in groups that received zein 3 or 5 days before the injuries had a pattern more similar to that found in intact skin. On the other hand, the group that received zein 7 days before injuries had collagen fibers in the neodermis arranged more similar to the control scarred skin. In addition, 40 days after injury, the papillary dermis was well organized in animals that received zein 3 or 5 days before skin injuries (Figure 3D).

**Figure 4:**
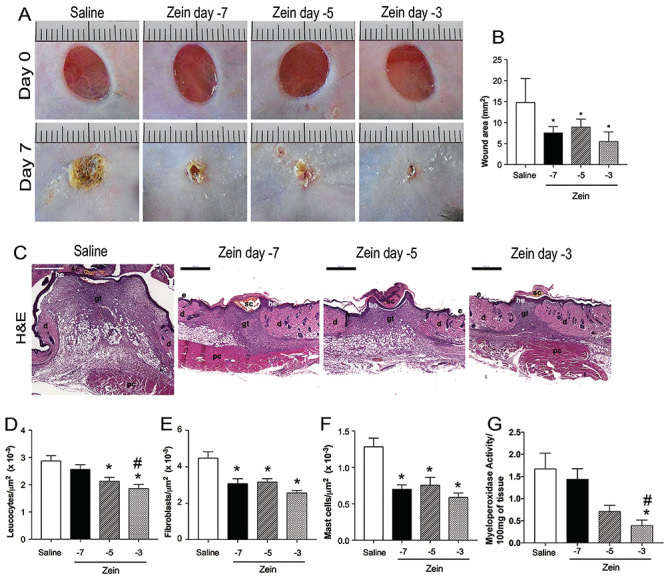
Subcutaneous injection of zein before skin injury reduced the inflammatory infiltrate and accelerated wound closure and scab drop. **A**, Macroscopic photographs of wounds immediately after skin injury (33 mm^2^) and 7 days after injury in mice injected with saline or zein plus adjuvant. **B**, Wound area 7 days after skin injury in mice injected with saline or zein + adjuvant at 7, 5, or 3 days before wounds. Data are reported as means±SE (n=6 mice, 12 wounds). *P≤0.05 compared with the saline group. **C**, Representative images of skin at day 7 after injury in the control mice (saline) and mice injected with zein at 7, 5, or 3 days before skin injury (H&E-stained sections). e, epidermis; d, dermis; gt, granulation tissue; he, hypertrophic epidermis; pc, panniculus carnosus; sc, scab. Scale bars: 500 µm. **D**-**F**, Morphometric analyses of leukocytes, fibroblasts, and mast cells. **G**, Quantification of myeloperoxidase activity. Control mice and mice injected with zein at 7, 5, or 3 days before injury. Data are reported as means±SE (n=6). *P≤0.05 compared with the saline group, ^#^P≤0.05 compared with the zein -7 group (ANOVA).

Macroscopic analysis of the wounds 7 days after injury and qualitative analysis of H&E-stained sections showed that, although re-epithelization was complete in all groups, the increase in the time interval between zein injection and skin injuries decreased its anti-inflammatory effects (Figure 4A and C). In mice injected with zein 5 or 7 days before skin injury, the scabs were more prominent and more firmly attached to the skin than in mice injected with zein 3 days before skin injury (Figure 4A). In turn, treatment with zein had a positive effect on wound closure regardless of the interval between treatment and injury (Figure 4B).

**Figure 5:**
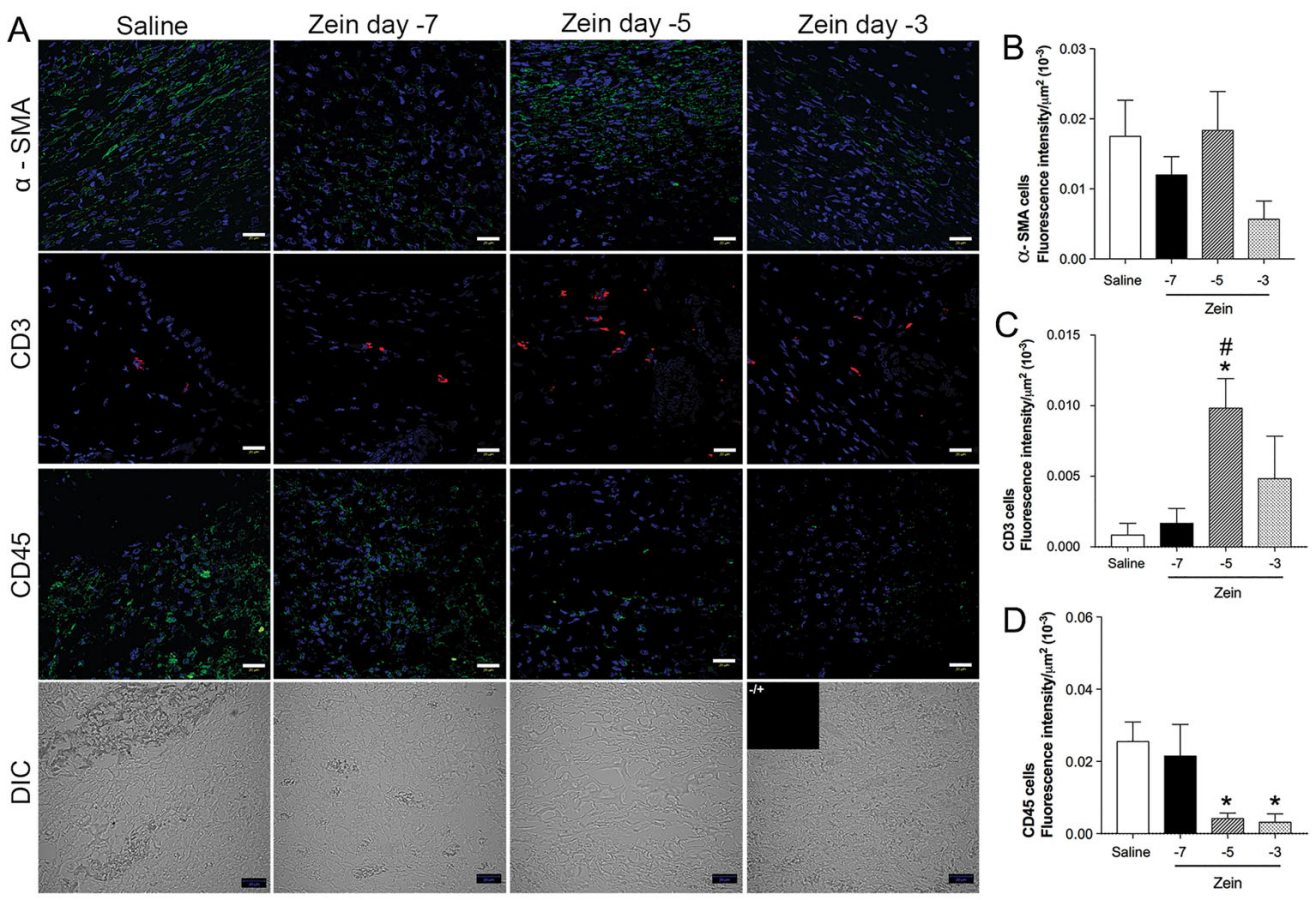
Subcutaneous injection of zein before skin injury reduced leukocyte number and increased T cells in the wound bed. **A**, Representative images of skin after immunostaining with anti-α-SMA (myofibroblasts), anti-CD3 (lymphocytes), or anti-CD45 (leukocytes) and nuclear counterstaining with 4'6-diamidino-2-phenylindol (blue), 7 days after skin injury. The bottom row shows typical sections of each group imaged using differential interference contrast (DIC) and an insert with a picture of control staining lacking the primary antibody. Scale bars: 20 µm. Fluorescence intensity in sections immunostained with anti-α-SMA (**B**), anti-CD3 (**C**), or anti-CD45 (**D**). Control mice and mice injected with zein at 7, 5, or 3 days before injury. Data are reported as means±SE (n=6). *P≤0.05 compared with the saline group, ^#^P≤0.05 compared with the zein -7 group (ANOVA).

Morphometric analysis of cellular infiltration in the granulation tissue showed that this treatment reduced the number of fibroblasts (Figure 4E) and mast cells (Figure 4F), regardless of the time of zein injection. On the other hand, morphometric evaluation of leukocytes in H&E-stained skin samples (Figure 4D) and immunofluorescence of skin sections incubated with anti-CD45 antibody (Figure 5) showed that zein injection on either day 3 or day 5 before injuries, but not on day 7, reduced the number of leukocytes in the wound bed. Figure 5 shows that the fluorescence intensity of α-SMA was not significantly altered by injection of zein on days 3, 5, or 7 before skin injuries.

Since neutrophils are prominent inflammatory cells that infiltrate the wound bed soon after skin injury, we performed an additional experiment to verify if subcutaneous injection of zein before injuries blocked neutrophil infiltration. Figure 4G shows a significant decrease in MPO activity in the skin samples of mice injected with zein 3, but not 5 or 7 days, before injuries.

## Discussion

The development of therapeutic strategies to prevent complications in wound healing is of general interest, especially because the number of scheduled surgeries has increased greatly in recent years (32). We show here that it was possible to improve healing of skin injuries through one subcutaneous injection of zein at a site distant from the injury site. We also show that, although injection of zein 10 min before skin injury was highly effective in improving the pattern of collagen arrangement in the neodermis, the injection of zein 3 or 5 days before injury also had positive effects and resulted in better wound healing. However, when the interval between zein injection and skin injury was increased to 7 days, wound healing did not improve. Furthermore, injection of zein 7 days before wounding did not reduce the inflammatory infiltrate and did not increase the number of T lymphocytes in the wound bed, but did reduce the number of mast cells. Our results suggest that reduction of mast cell numbers by itself was not sufficient to significantly ameliorate the pattern of collagen deposition in neodermis. Our results agree with those showing that reduction in the number of mast cells does not significantly alter skin wound healing (33,34). However, the accurate determination of mast cells roles during healing are challenged by the pleiotropic and redundant effects of their mediators, which can affect healing even in low quantity.

Injection of zein had a positive effect on wound closure, independently of the time interval between zein injection and skin injury (Figure 4B). However, the effects of zein on wound closure did not correlate with the effects of zein on myofibroblast number (Figure 5B). These results suggest that the injection of zein had positive effects on wound closure that was independent from the number of myofibroblasts in the granulation tissue. It is interesting to note that wound closure in rodents is greatly promoted by contraction of the panniculus carnosus, a thin layer of muscular tissue present in the subcutis, which is virtually absent in humans (35). Further experiments would be necessary to verify if the effects of zein on wound closure occurred via contraction of panniculus carnosus, especially using another model of skin wounding that permits distinguishing the action of panniculus carnosus from the action of myofibroblasts on skin wound closure.

The improved extracellular matrix reorganization in the neodermis of mice that received zein 3 or 5 days before skin injury also cannot be directly correlated with a significant change in the number of fibroblasts in the granulation tissue. However, the positive effect of zein on collagen pattern correlated with the lower number of neutrophils in the wound bed. Reduced numbers of neutrophils may impact on the extension and quality of scarring as these cells produce proteases and reactive oxygen species that may increase initial tissue damage (36,37).

Recent studies have shown increased numbers of T lymphocytes at injured sites in various organs and it is proposed that T lymphocytes are involved in making the injured environment more conducive to regeneration (38). It has been suggested that T lymphocytes located near hair follicles are important for hair follicle biology and skin regeneration (39). During cutaneous healing, T lymphocytes increase in the wound bed peaking on day 7 after injury (7) and Treg cells may limit neutrophil infiltration and facilitate skin wound healing (8,40). In agreement with the present work, we have previously shown an increase in T-lymphocyte numbers in the skin wound bed in OVA- or zein-tolerant mice that received an intraperitoneal injection of these proteins minutes before injury (28,29). It is possible that the increase in the numbers of T lymphocytes in the wound bed of mice injected with the orally-tolerated protein zein contributed to the resolution of inflammation and to reducing fibrosis through changes in the activation of fibroblasts and/or myofibroblasts. At this time, it is not possible to discern if the change in collagen pattern after zein injection was due to collagen remodeling or to a change in collagen deposition. To further characterize the role of T cells in skin wound healing after tolerized protein injection, it would be interesting to identify the T cell subtypes in the skin lesions; however, flow cytometry analysis rather than *in situ* immunofluorescence is indicated for this purpose, as the number of T cells in the wound bed is low.

In conclusion, the systemic effects of subcutaneous injection of zein in mice fed with chow containing maize lasted for at least 5 days, reduced the inflammatory infiltrate in the wound bed, and promoted better wound healing. Our data indicated that the systemic effects of oral tolerance may improve cutaneous wound healing when skin injury occurs within a 5-day interval after subcutaneous injection of an immunologically-tolerated protein. These data are quite important for future clinical applications in scheduled surgeries. Zein is the most abundant protein of maize, one of the most popular cereal grains in the world, and allergy to zein is not common.
